# Biliary Calculi—Convulsions and Death

**Published:** 1870-03

**Authors:** J. A. Ballard

**Affiliations:** Chicago, Ill.


ARTICLE X.

### BILIARY CALCULI—CONVULSIONS AND DEATH.

BY J. A. BALLARD, M.D., Chicago, Ill.

The following case came under my observation in Dec., 1869, and I have thought it of sufficient interest to report:—

Was called to see the patient, a man 37 years of age, at 10 o'clock on the morning of the 25th. He was a strong, robust looking man, who had been much addicted to the use of alcoholic stimulants. I found him suffering slight pain, which he referred to the region of the stomach: the pain had been more severe, but had partially subsided. I prescribed tr. opii, in 25 drop doses. At 12.30 P.M., pain came on very severe; gave morph. sulph. in one-third-grain doses. Sharp pain came in paroxysms, with a dull, heavy pain in the intervals. At 2 o'clock the pain was so severe as to cause the patient to writhe and groan with agony. He exhibited considerable anxiety, and at times was a little delirious. Continued anodyne and applied warm fomentations to epigastric region, with the effect of relieving the pain in a few minutes.

After half an hour, left the patient feeling quite comfortable, and gave directions for continuing the morph. powders. Saw him again about 5 o'clock, and found that in my absence he had had two severe attacks of pain, and in the last went into convulsions; there had been nausea, vomiting, and faintness, and now the patient was much prostrated; pain severe in epigastric region, shooting to the back and up to the right shoulder, these symptoms strongly indicating the passage of biliary calculi. I now gave chloroform, in half-drachm doses, and continued warm fomentations. Patient slept considerable during the night, but at times was delirious. *26th.* Patient is considerably under the influence of anodynes, and takes but little nourishment. Dull, heavy pain continues, with sharp pain at times, and more or less delirium.

Saw the patient at 9 o'clock P.M. He had just suffered for a few minutes from severe pain; still referred to the epigastrium. The patient was now quite delirious, and I saw that he was failing rapidly. Gave spirits amm. aromat., in drachm doses, and chloroform, 20 minims every hour and a-half, and ordered hot and stimulating applications to feet and legs. Patient grew more delirious, complaining of the pain at times, and rapidly sunk till 2 o'clock A.M., morning of the 27th, when he expired.

In the afternoon, assisted by Dr. Croskey, I made a *post mortem* examination. After laying open the subject, upon taking the gall-bladder in my hand, much to my satisfaction, I found that it contained calculi. Removing the bladder and laying it open disclosed to our eyes 25 biliary calculi; some of them five-eighths of an inch in diameter, and the smallest nearly two-eighths in diameter. Six of these calculi were lodged in the *cystic duct* and *ductus communis choledochus;* and so firmly were they impacted as to require considerable pressure to force them through the orifice into the duodenum. The gall-bladder was not distended with bile.

There was a large deposit of fat upon the subject. The walls of the heart were found to be in a state of fatty degeneration, at one point of the right ventricle, the muscular tissue not being thicker than a wafer, such as formerly used for sealing letters. The mucous membrane of the stomach was somewhat inflamed. These latter morbid conditions undoubtedly being due to the long-continued use of alcholic stimulants; and to what extent the formation of the calculi was due to the same cause, by producing functional disorder of the liver, I am unable to say. The liver was of about normal size, perhaps a trifle larger, and slightly friable.

